# Biogeography of Virioplankton Abundance and Subcluster Patterns in the Northwest Pacific: A Large‐Scale Perspective

**DOI:** 10.1002/mbo3.70161

**Published:** 2025-11-21

**Authors:** Yuan Zhao, Yanchu Zhao, Yi Dong, Xiaoxia Sun, Wuchang Zhang, Li Zhao, Gérald Grégori

**Affiliations:** ^1^ Laboratory of Marine Ecology and Environmental Sciences, Institute of Oceanology Chinese Academy of Sciences Qingdao China; ^2^ Laboratory for Marine Ecology and Environmental Science Qingdao Marine Science and Technology Center Qingdao China; ^3^ Center for Ocean Mega‐Science Chinese Academy of Sciences Qingdao China; ^4^ Center of Eco‐environmental Monitoring and Scientific Research, Administration of Ecology and Environment of Haihe River Basin and Beihai Sea Area Ministry of Ecology and Environment of People's Republic of China Tianjin China; ^5^ Jiaozhou Bay Marine Ecosystem Research Station, Institute of Oceanology Chinese Academy of Sciences Qingdao China; ^6^ Aix‐Marseille University, Toulon University, CNRS, IRD Mediterranean Institute of Oceanography UM110 Marseille France

**Keywords:** abundance, Northwest Pacific, phenotypic diversity, picoplankton, viral subclusters, virioplankton

## Abstract

Marine virioplankton, the most abundant biological entities in the ocean, play essential roles in microbial ecology and biogeochemical cycling. This study investigates their biogeography in the Northwest Pacific using enhanced‐resolution flow cytometry and phenotypic diversity analyses. By resolving four consistent viral subclusters across oceanic and coastal waters and detecting a fifth subcluster in the Yellow Sea, we revealed previously unrecognized patterns of viral community structures. Viral abundances ranged from 3.69 × 10⁶ to 17.09 × 10⁶ particles/mL, showing clear coastal‐oceanic differentiation. Environmental gradients, particularly temperature, chlorophyll, and picoplankton abundance, emerged as the primary drivers of virioplankton community structure. These findings underscored the tight coupling between viral populations and their microbial hosts across contrasting marine environments. Phenotypic diversity analysis revealed distinct viral communities in the Luzon Strait, despite comparable abundance patterns to adjacent regions, demonstrating the method's sensitivity in detecting subtle community shifts. This study advances understanding of marine viral biogeography and introduces a robust framework for investigating viral community dynamics. The approach enables high‐throughput screening across large spatial scales while maintaining sensitivity to fine‐scale community variations, offering new possibilities for monitoring viral responses to environmental change in marine ecosystems.

## Graphical abstract

Enhanced‐resolution flow cytometry combined with phenotypic diversity analysis reveals four consistent viral subclusters and a fifth restricted to the Yellow Sea across the Northwest Pacific. These results identify a cryptic diversity hotspot in the Luzon Strait and demonstrate how environmental and biological gradients shape large‐scale patterns in marine viral biogeography.

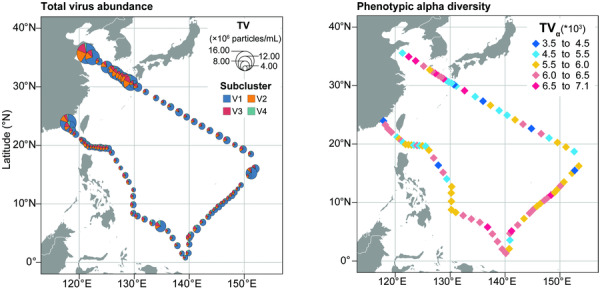

## 1 Introduction

Marine virioplankton, the viral components of marine planktonic communities, are ubiquitous and overwhelmingly abundant across the global ocean. Estimates place their concentration between 10⁴ and 10⁸ particles per milliliter of seawater (Wommack and Colwell 2000), adding up to approximately 10³⁰ virus‐like particles (VLPs) globally (Cobián Güemes et al. 2016). Despite their small physical size and relatively minor contribution to total marine biomass ( ~30 Megatons of carbon, or ~1% of total marine biomass) (Bar‐On and Milo 2019), virioplankton exert disproportionately large effects on marine ecosystem functioning.

Viruses play integral roles in regulating microbial food webs by selectively infecting and lysing their hosts, thus influencing microbial population dynamics, community composition, and turnover rates (Middelboe and Brussaard 2017), often through the “kill‐the‐winner” mechanism where the most abundant hosts face the highest viral pressure (Thingstad 2000). Lysis of microbial hosts by viruses leads to the release of cellular contents into the dissolved organic matter pool, fueling microbial loop processes and influencing carbon and nutrient cycling in the ocean (Jover et al. 2014). Additionally, virus can also modulate host metabolic pathways by introducing auxiliary metabolic genes (AMGs) that enhance processes such as photosynthesis and nitrogen cycling (Rosenwasser et al. 2016). These diverse interactions position marine viruses as critical agents in biogeochemical transformations and microbial ecological networks.

Given their ecological importance, accurate quantification of marine viruses is essential for understanding microbial interactions and ecosystem processes. Flow cytometry (FCM) provides a powerful means of assessing viral abundance and population structure by detecting fluorescently stained particles in seawater. Since its first application to marine viral research in the late 1990s (Marie et al. 1999), FCM has enabled rapid and reproducible measurements of VLPs across diverse aquatic environments, greatly improving our capacity to evaluate virus–host relationships and spatial variability.

Typical FCM analyses using blue laser (488 nm) and nucleic acid stains such as SYBR Green I or SYBR Gold reveal multiple clusters of viral signals that differ in fluorescence intensity and light scatter, reflecting variations in particle size and nucleic acid content. Most oceanic studies have reported two to three dominant viral groups (Liang et al. 2017; Magiopoulos and Pitta 2012; Payet and Suttle 2008; Wei et al. 2018; Yang et al. 2014), while additional minor clusters occasionally appear under specific environmental conditions (Baudoux et al. 2008; Mojica et al. 2016). However, such minor clusters are rare and often limited to specific marine regions or transient ecological events.

Over the past decade, several studies have explored large‐scale patterns of marine viral communities, revealing distinct biogeographic trends across different water masses and depth layers (De Corte et al. 2016; Liang et al. 2014; Liang et al. 2017; Mojica et al. 2016; Yang et al. 2010; Yang et al. 2014). Most of these studies, however, concentrated on oceanic settings, whereas the mechanisms driving viral community transitions from coastal to offshore waters, where environmental gradients are especially strong, remain poorly characterized.

Recent advances in flow cytometric instrumentation, particularly the addition of 405 nm violet lasers and highly sensitive avalanche photodiode detectors (APD), have greatly improved signal resolution for detecting nano‐sized viral particles (Zhao et al. 2023). These developments allow clearer separation of viral signals from background noise and more precise delineation of viral subclusters. Using this improved configuration, Zhao et al. (2020) reported four distinct viral subclusters in the euphotic zone and three subclusters below the euphotic zone in the vicinity of Caroline Seamount in the Western Pacific. More recent observations in nearshore waters revealed even higher complexity, with up to six distinguishable subclusters (Zhao et al. 2023). This pronounced spatial heterogeneity in subcluster patterns likely reflects substantial variation in viral community structure between oceanic and coastal environments.

**Figure 1:**
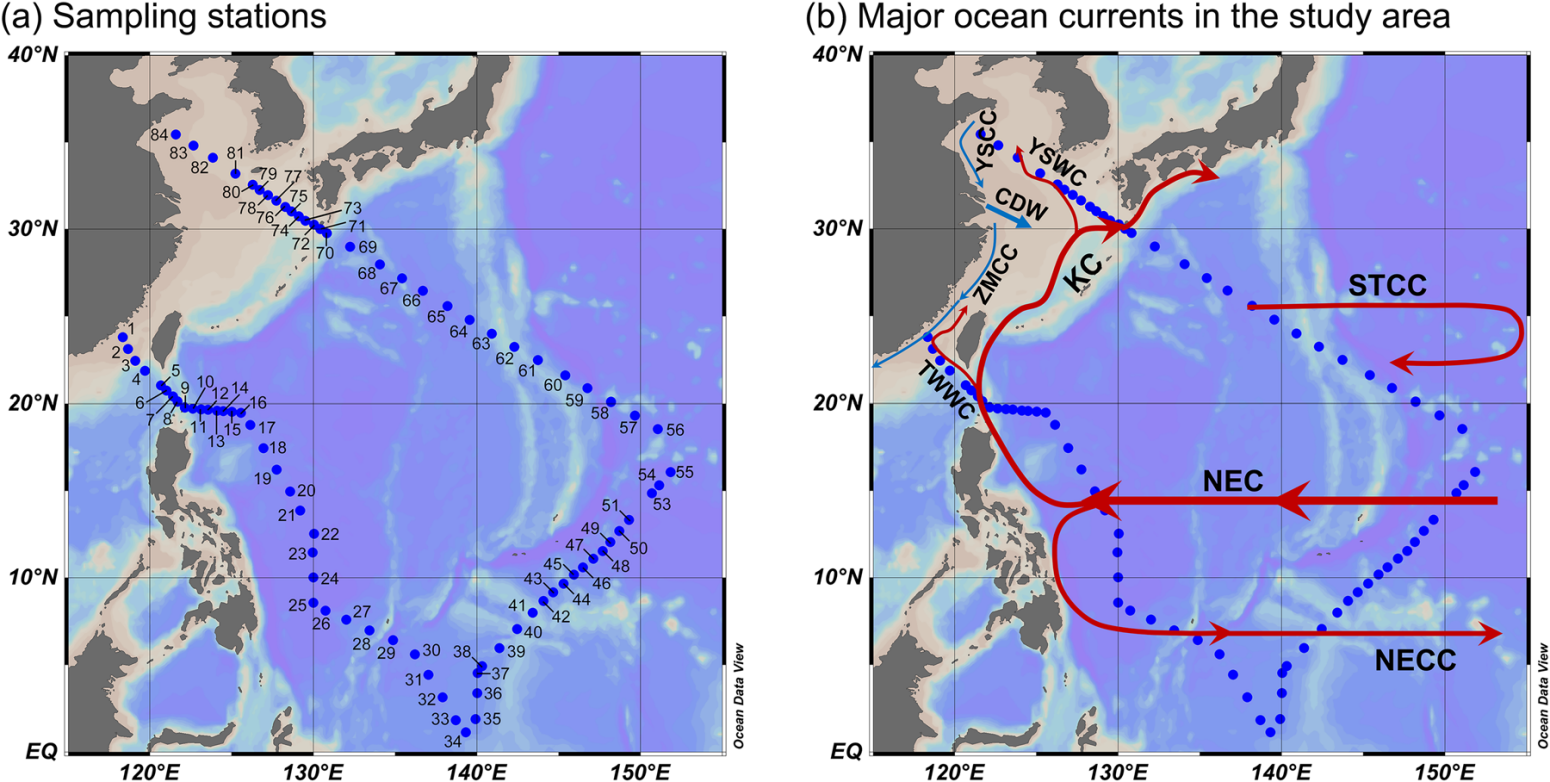
Sampling stations and major ocean currents in the study area. (a) Map showing the sampling stations in this study. (b) The major ocean currents in the study area. CDW, Changjiang Diluted Water; KC, Kuroshio Current; NEC, North Equatorial Current; NECC, North Equatorial Countercurrent; STCC, Subtropical Countercurrent; TWWC, Taiwan Warm Current; YSCC, Yellow Sea Coastal Current; YSWC, Yellow Sea Warm Current; ZMCC, ZheMin Coastal Current.

Despite the growing number of studies on marine viral biogeography, most have focused on open‐ocean systems, providing limited understanding of how viral community composition transitions along the coastal–oceanic continuum. In addition, the relatively coarse resolution of earlier flow cytometric approaches has constrained efforts to resolve fine‐scale phenotypic variability among viral populations. To address this knowledge gap, we conducted a comprehensive survey across the Northwest Pacific, spanning a continuous transect from coastal to oceanic waters (Figure 1). This region provides an ideal natural setting for investigating viral biogeography, as it encompasses multiple interacting current systems and distinct water masses. The objectives of this study were to (1) characterize spatial changes in viral abundance and subcluster composition along the coastal‐oceanic continuum, (2) examine the relationship between viral distribution patterns and key environmental and biological variables, and (3) identify the main environmental drivers of viral community structure. Through this approach, we aim to elucidate the spatial dynamics of marine virioplankton across contrasting ecosystems and provide new insights into their ecological roles in diverse marine environments.

## 2 Materials and Methods

### 2.1 Study Area and Sampling

A survey was conducted in the Northwest Pacific aboard R.V. “*Kexue*” from March 14 to April 18, 2018. The transect spanned the Jiulong River Estuary, the Luzon Strait, the equatorial Pacific, the North Pacific Subtropical Gyre, and the offshore waters of the Yellow Sea, covering 84 sampling stations. Unfortunately, the sample from Stn. 52 was lost during transit. Sampling station locations and major currents in the study area are shown in Figure 1.

Surface water samples (5 m depth) were continuously collected through the ship's underway sampling system. For virioplankton and picoplankton analyses, 4‐mL aliquots were fixed with glutaraldehyde (1% final concentration, v/v) for 30 min in darkness at room temperature and then flash‐frozen in liquid nitrogen until laboratory analysis (Marie et al. 1999).

Surface water temperature (°C), salinity, and in situ chlorophyll fluorescence were recorded using a Thermosalinograph (SBE 38, SeaBird Scientific, USA) integrated with the underway sampling system. Nutrients (nitrate, phosphate, and dissolved silicate) and net primary production (NPP) were obtained from the Copernicus Global Ocean Biogeochemistry Hindcast data set with a daily resolution of 0.25° × 0.25° (CEMES, https://doi.org/10.48670/moi‐00019).

### 2.2 Virioplankton and Picoplankton Enumeration

Both virioplankton and picoplankton were enumerated using FCM. Before analysis, samples were thawed in the dark and gently mixed, then passed through a 100‐µm mesh to remove debris and large particles.

**Figure A1:**
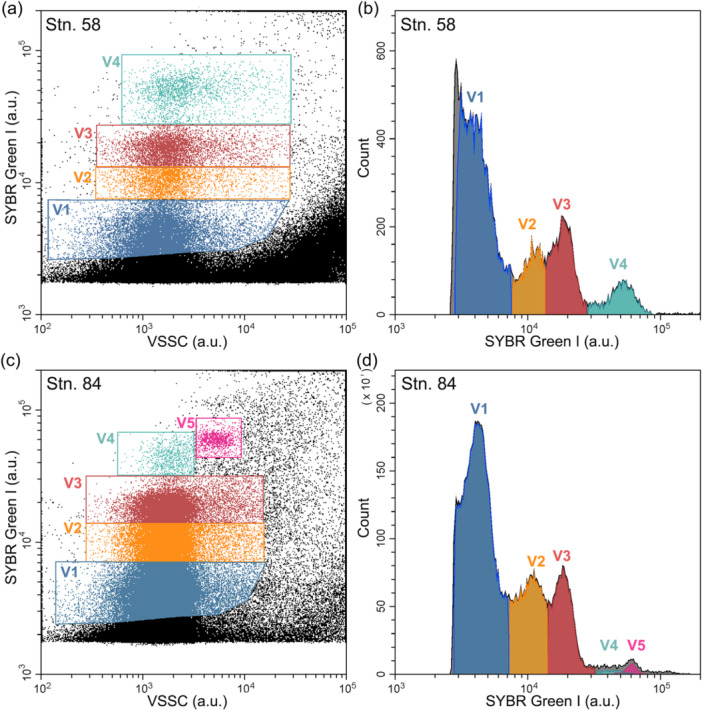
Cytograms of virioplankton subclusters obtained in this study. (a) and (b) Cytograms from Stn. 58, illustrating the division of viral populations into four subclusters (V1, V2, and so on), with subcluster numbers increasing with fluorescence intensity. (c) and (d) Cytograms from Stn. 84, showing the separation of viral populations into five subclusters, following the same classification scheme. a.u.: arbitrary units.

For virioplankton, a Cytoflex S flow cytometer (Beckman Coulter, USA) equipped with blue (488 nm) and violet (405 nm) lasers was used following the general procedure of Zhao et al. (2023). Samples were diluted fivefold in filtered, autoclaved TE buffer (100 mM Tris‐Cl, 10 mM EDTA, pH 8.0; Sigma‐Aldrich, USA), stained with SYBR Green I (Molecular Probes Inc., 5 × 10^−^^5 ^v/v final concentration of the commercial stock), heated at 80°C for 10 min, and cooled for 5 min. Detection of violet side scatter (VSSC) signals enhanced the discrimination of small viral particles. Four to five viral subclusters were identified based on VSSC versus SYBR Green I fluorescence intensities (Figure A1). Total VLP abundance was the sum of all viral subcluster abundance.

Picoplankton, including the photosynthetic *Synechococcus* (SYN), *Prochlorococcus* (PRO), picoeukaryotes (PEUK), and the heterotrophic prokaryotes (HP, i.e., bacteria and archaea), were enumerated using a FACSJazz flow cytometer (Becton Dickinson, USA) following Marie et al. (2000). Two‐micrometer fluorescent beads (Polysciences, USA) served as internal standards. Autotrophic picoplankton (SYN, PRO, and PEUK) were distinguished by their scatter properties and chlorophyll a/phycoerythrin autofluorescence. For HP, samples were diluted sixfold with TE buffer and stained with SYBR Green I (10^−^^4 ^v/v final concentration) and incubated for 20 min in the dark at room temperature. Based on green fluorescence and side scatter intensity, HP populations were separated into high nucleic acid content heterotrophic prokaryotes (HNA) and low nucleic acid content heterotrophic prokaryotes (LNA) (Gasol and Del Giorgio 2000).

### 2.3 Phenotypic Diversity Analyses

The bio‐optical characteristics of individual cells/particles obtained from FCM measurements can be utilized to compute biodiversity indices (Li 1997). Raw FCM data (.fcs) was extracted and processed to estimate phenotypic traits such as morphology and nucleic acid content of VLPs as described by Props et al. (2016). The data were processed using the R package “*Phenoflow*” v1.1.2 (Props et al. 2016).

For alpha diversity analysis, phenotypic intensity values were normalized to [0,1] interval using the maximum FL‐1 H (SYBR Green I fluorescence) intensity. The inverse Simpson index (D2) was computed using the “Diversity_rf()” function on a refined 256 × 256 grid for kernel density estimation using FL‐1 H and VSSC H. Beta diversity was assessed using Bray‐Curtis dissimilarities of phenotypic feature vectors. Principal Coordinates Analysis (PCoA) and permutational multivariate analysis of variance (PERMANOVA, “Adonis()” function in R package *“vegan”*) were applied to visualize phenotypic diversity patterns and test for significant group differences.

### 2.4 Statistical Analyses and Visualization

FCM data were analyzed using CytExpert software (Beckman Coulter, USA) for virioplankton and Summit software (Dako Colorado, USA) for picoplankton. Maps of study regions, surface temperature, salinity, in situ chlorophyll fluorescence, and temperature‐salinity (T‐S) diagram were created using Ocean Data View (ODV) (Schlitzer 2022).

Spatial and community analyses (PCoA, PERMANOVA, Mantel, and RDA) were performed in R (v4.4.1) (R Core Team 2020) using packages “*vegan*” v2.6‐8 (Oksanen et al. 2007), “*pairwiseAdonis*” v0.4.1 (Martinez Arbizu 2020), “*linkET*” v0.0.7.4 (Huang 2021), “*ggplot2*” v3.5.1 (Wickham 2011), “*ggordiplots*” v0.4.3 (Quensen et al. 2024), “*ggrepel*” v0.9.6 (Slowikowski 2023), and others as cited below.

Geographic distribution patterns of the virioplankton and picoplankton were analyzed using PCoA and PERMANOVA (Adonis) based on Bray‐Curtis dissimilarity matrices. Standard deviation‐based confidence ellipses were drawn with the “gg_ordiplot()” function from the package “*ggordiplots*” to visualize group confidence areas (*n* ≥ 3); the single‐station estuarine region (*n* = 1) was manually circled. Pairwise Adonis tests (999 permutations) with Benjamini‐Hochberg correction were used to compare groups. Mantel tests examined correlations between virioplankton abundance, phenotypic diversity, and environmental/biological factors. Redundancy analysis (RDA) determined associations between virioplankton metrics and environmental/biological factors. Environmental Fit (“envfit” function in R package “*vegan*”) was applied to PCoA ordination to evaluate the influence of environmental factors on viral beta diversity. Significance was tested using 999 permutations. Where required, data were log‐transformed (base 10) to meet statistical assumptions.

All visualizations were created in R‐Studio (v2024.09.0 + 375) (RStudio Team 2020) using R packages “*ggplot2*” v3.5.1 (Wickham 2011), “*scatterpie*” v0.2.4 (Yu 2024), “*rnaturalearth*” v1.0.1 (Massicotte and South 2023), “*rnaturalearthdata*” v1.0.0 (South et al. 2024), “*tidyr*” v1.3.1 (Wickham et al. 2024), and “*dplyr*” v1.1.4 (Wickham et al. 2023).

## 3 Results

### 3.1 Environmental Variables

**Figure 2:**
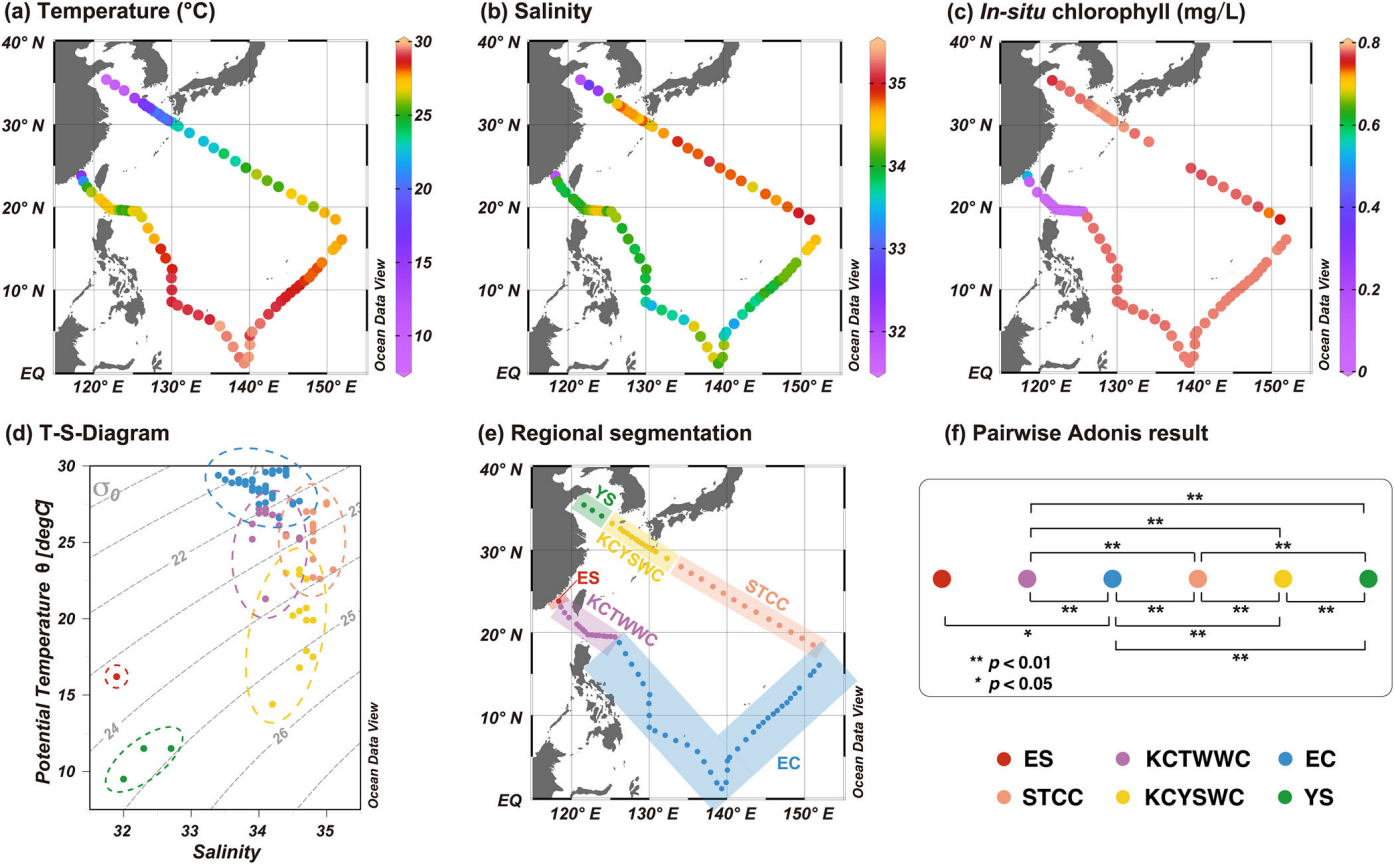
Spatial distribution of environmental parameters and regional classification in the Northwest Pacific. (a) temperature (°C); (b) salinity; (c) in situ chlorophyll (mg/L); (d) T‐S diagram; (e) regional segmentation of the study area showing ES (Jiulong River Estuary), KCTWWC (Kuroshio Current‐Taiwan Warm Current), EC (Equatorial Current), STCC (Subtropical Countercurrent), KCYSWC (Kuroshio Current‐Yellow Sea Warm Current), and YS (Yellow Sea) regions; (f) Pairwise Adonis analysis result showing statistical differences in environmental parameters between regions. Statistically significant differences between regions were marked as *(*p* < 0.05) or **(*p* < 0.01). Colored dots in (d–f) represent data set from different regions.

This study encompassed diverse environmental settings, spanning nearshore, subtropical, and equatorial regions in the Northwest Pacific. Surface water temperature averaged 25.53°C ± 4.56°C, ranging from 9.5°C in the Yellow Sea to above 28.5°C in the equatorial region (Figure 2a, Table 1). Salinity exhibited a mean of 34.17 ± 0.59, with the lowest value of 31.9 recorded in the Jiulong River Estuary and values exceeding 35 in the Subtropical Countercurrent‐affected region (Figure 2b). *In situ* chlorophyll remained relatively stable throughout the survey (mean: 0.64 ± 0.29 mg/L), with notably lower values observed in the Jiulong River Estuary and Luzon Strait (Figure 2c).

**Table 1:** Environmental variables across different regions of the Northwest Pacific.

	All stations	ES	KCTWWC	EC	STCC	KCYSWC	YS
Temperature	25.53 ± 4.56	16.20	26.07 ± 1.53	28.69 ± 0.75	25.37 ± 1.77	19.96 ± 2.69	10.83 ± 1.15
Salinity	34.17 ± 0.59	31.90	34.17 ± 0.22	34.02 ± 0.29	34.82 ± 0.16	34.6 ± 0.16	32.33 ± 0.35
Chl	0.64 ± 0.29	0.53	0.02 ± 0.03	0.78 ± 0.00	0.77 ± 0.01	0.78 ± 0.00	0.77 ± 0.00
NPP	5.08 ± 4.93	10.20	7.32 ± 4.72	2.49 ± 1.74	3.36 ± 2.58	12.21 ± 5.76	1.46 ± 0.35
NO3	0.07 ± 0.31	2.20	0.00 ± 0.00	0.00 ± 0.00	0.00 ± 0.00	0.06 ± 0.06	1.10 ± 0.10
PO4	0.06 ± 0.06	0.00	0.06 ± 0.05	0.09 ± 0.07	0.01 ± 0.03	0.05 ± 0.05	0.00 ± 0.00
Si	2.09 ± 1.34	7.10	1.93 ± 0.50	1.85 ± 0.17	1.50 ± 0.16	1.88 ± 0.54	7.73 ± 2.34

*Note:* Values are presented as mean ± standard deviation. Abbreviations: °C, temperature; Chl, in situ chlorophyll fluorescence, mg/L; EC, equatorial current; ES, Jiulong River Estuary; KCTWWC, Kuroshiocurrent‐Taiwan warm current; KCYSWC, Kuroshio current‐Yellow Sea warm current; NPP, net primary production, μg/L·d; NO3, nitrate, PO4, Phosphate, Si, dissolved silicate, μmol/L; STCC, subtropical countercurrent; YS, Yellow Sea.

**Figure A2:**
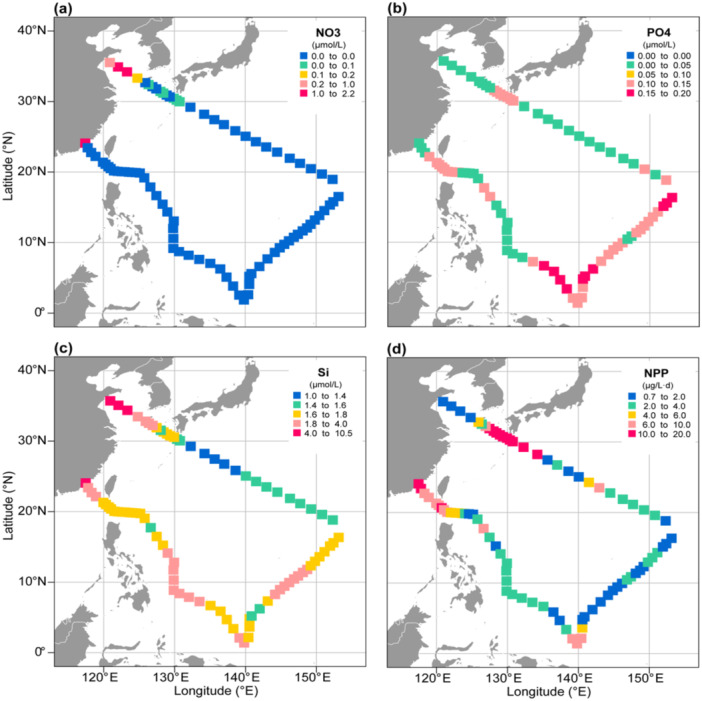
Spatial distribution of nutrient concentration and net primary productivity in the surface waters of the Northwest Pacific. (a) NO3, Nitrate concentration, μmol/L; (b) PO4, phosphate concentration, μmol/L; (c) Si, dissolved silicate concentration, μmol/L; (d) NPP, net primary production, μg/L·d. Data was obtained from the Copernicus Global Ocean Biogeochemistry Hindcast data set with 0.25° × 0.25° daily resolution.

The spatial distribution of biogeochemical variables revealed distinct patterns across the study area (Figure A2). NPP peaked in regions influenced by the two Kuroshio branches, namely the Taiwan Warm Current and the Yellow Sea Warm Current. Nitrate concentrations were elevated in offshore waters but depleted in the pelagic zones, whereas phosphate showed an opposite trend. Dissolved silicate concentrations were highest in the estuary and Yellow Sea, with minimal values in the STCC‐influenced region.

The temperature‐salinity (T‐S) diagram (Figure 2d) differentiated six hydrographic regions within the study area (Figure 2e): the Jiulong River Estuary (ES), the Kuroshio Current‐Taiwan Warm Current (KCTWWC), the Equatorial Current (EC), the Subtropical Countercurrent (STCC), the Kuroshio Current‐Yellow Sea Warm Current (KCYSWC), and the Yellow Sea (YS). Mean values of key environmental variables for each region are summarized in Table 1. Pairwise Adonis analysis of environmental variables (temperature, salinity, chlorophyll, NPP, and nutrients) revealed significant differences among regions (Figure 2f). The ES region, represented by a single sampling station, was characterized by low salinity, high nutrient concentrations, and enhanced productivity. However, owing to limited sampling, significant differences were only detected between the ES and EC regions.

### 3.2 Abundance and Community Structure of Virioplankton

FCM analyses revealed four well‐defined viral subclusters, designated as V1–V4, which were prevalent in the surface layer of the Northwest Pacific. These subclusters exhibited broadly similar values for VSSC, indicating comparable size and morphology. However, they displayed distinct SYBR Green I fluorescence intensities, reflecting differences in nucleic acid contents (Figure A1a,b). Notably, an additional subcluster, V5, characterized by higher VSSC and SYBR Green I fluorescence intensity (Figure A1c,d), was uniquely observed at Stn. 84 in YS. Because of its localized occurrence, V5 was merged with V4 for subsequent statistical analyses at this station.

**Figure 3:**
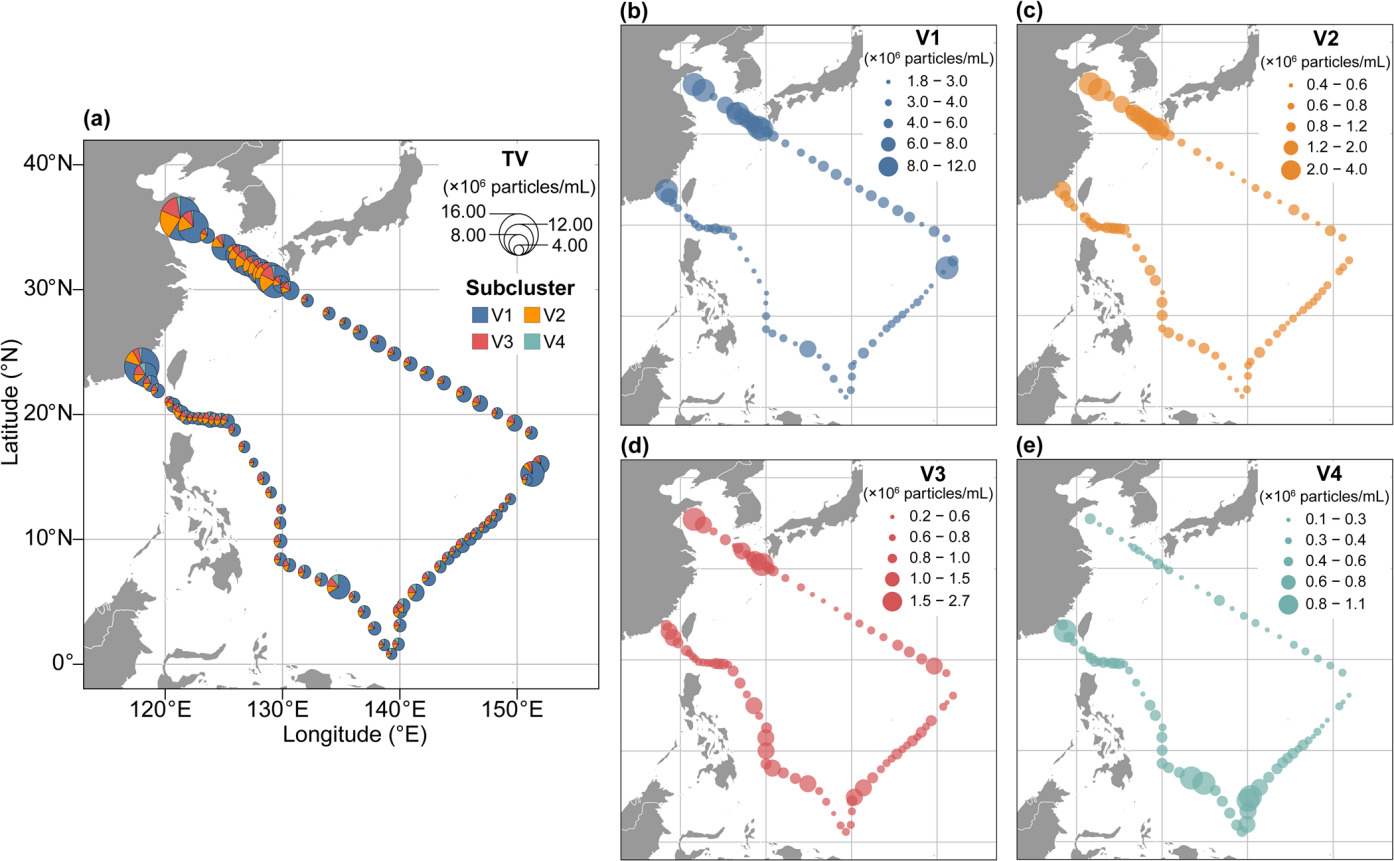
Spatial distribution of viral abundance in the surface waters of the Northwest Pacific. (a) Total VLP (TV) abundance, that is, the sum of V1–V4 abundance. (b–e) Abundance of individual viral subclusters (V1–V4). See Figure A2 for details on the abundance and percentage of viral subclusters in panel (a).

**Figure A3:**
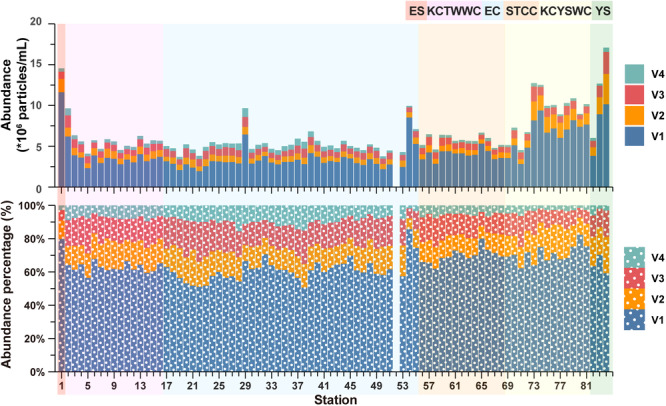
Details of abundance and percentage of viral subclusters across survey stations. Background colors represent different regions.

Total VLP (TV) abundance ranged from 3.69 × 10^6^ particles/mL to 17.09 × 10^6^ particles/mL, with a mean value of 6.37 ± 2.56 × 10^6^ (Table 2). Spatial patterns revealed a clear coastal‐oceanic gradient (Figure 3a, Figure A3), with higher abundances in coastal regions (ES: 14.55 × 10⁶, KCYSWC: 9.08 × 10⁶, YS: 11.92 × 10⁶ particles/mL) compared to oceanic regions (KCTWWC: 5.66 × 10⁶, EC: 5.30 × 10⁶, STCC: 5.71 × 10⁶ particles/mL). Standard deviations indicated greater variability in coastal regions, especially in the YS and KCYSWC, than in oceanic regions such as the STCC (Table 2).

**Table 2:** Abundance and composition of virioplankton and picoplankton communities across different regions of the Northwest Pacific.

	All stations	ES	KCTWWC	EC	STCC	KCYSWC	YS
TV	6.37 ± 2.56	14.55	5.66 ± 1.26	5.30 ± 1.27	5.71 ± 0.65	9.08 ± 2.54	11.92 ± 5.57
V1	4.20 ± 2.00	11.61	3.55 ± 0.86	3.28 ± 1.18	4.00 ± 0.61	6.46 ± 1.91	7.62 ± 3.35
V2	0.89 ± 0.49	1.63	0.83 ± 0.16	0.70 ± 0.11	0.64 ± 0.11	1.38 ± 0.57	2.29 ± 1.34
V3	0.86 ± 0.29	0.89	0.85 ± 0.21	0.82 ± 0.16	0.75 ± 0.14	0.95 ± 0.30	1.63 ± 0.97
V4	0.40 ± 0.16	0.41	0.41 ± 0.13	0.48 ± 0.18	0.31 ± 0.05	0.27 ± 0.07	0.37 ± 0.13
V1%	64.58 ± 7.03%	79.83%	62.63 ± 2.90%	61.03 ± 6.72%	69.84 ± 4.39%	70.81 ± 5.33%	64.37 ± 5.56%
V2%	13.89 ± 2.95%	11.21%	14.86 ± 2.01%	13.75 ± 2.77%	11.31 ± 1.75%	14.90 ± 3.31%	18.65 ± 2.79%
V3%	14.40 ± 3.52%	6.13%	15.13 ± 2.25%	15.97 ± 3.24%	13.32 ± 2.41%	10.98 ± 3.35%	13.30 ± 2.32%
V4%	7.10 ± 3.12%	2.82%	7.37 ± 1.17%	9.23 ± 2.79%	5.50 ± 1.10%	3.30 ± 1.30%	3.66 ± 2.08%
SYN	6.90 ± 11.91	17.25	8.43 ± 16.59	2.14 ± 4.24	2.42 ± 3.32	22.98 ± 13.84	5.86 ± 5.62
PRO	5.09 ± 8.87	2.14	3.58 ± 5.88	5.72 ± 10.78	1.05 ± 1.37	10.21 ± 9.02	0.84 ± 0.53
PEUK	4.36 ± 7.86	34.08	1.82 ± 1.33	1.07 ± 0.51	1.24 ± 0.53	12.33 ± 7.42	27.78 ± 13.80
HNA	5.07 ± 2.27	9.80	4.18 ± 1.12	4.44 ± 0.85	3.88 ± 0.33	7.52 ± 1.76	10.51 ± 7.28
LNA	2.38 ± 0.80	4.10	2.20 ± 0.72	2.08 ± 0.34	2.03 ± 0.28	3.28 ± 0.95	3.98 ± 1.09
VPR	8.74 ± 2.13	10.46	9.04 ± 1.41	8.28 ± 2.12	9.74 ± 1.60	8.50 ± 2.37	9.14 ± 5.16

*Note:* Values are presented as mean ± standard deviation. Abbreviations: HNA, high nucleic acid content heterotrophic prokaryotes, LNA, low nucleic acid content heterotrophic prokaryotes, ×10^5^ cells/mL; PRO, *Prochlorococcus*, PEUK, picoeukaryotes, ×103 cells/mL; SYN, *Synechococcus*; TV, total VLPs, ×106 particles/mL; V1–V4: viral subclusters (×106 particles/mL) and their relative abundance (%); VPR, virus to prokaryotes ratio. Region abbreviations as in Table 1.

Viral communities also exhibited significant regional variability in both abundance and relative subcluster composition (Figures 3a and A3). Subcluster V1, characterized by the lowest fluorescence intensity, dominated the viral community across all regions, reaching maximum abundance in ES (11.61 × 10⁶ particles/mL). Conversely, V4, with the highest fluorescence intensity, maintained the lowest abundance and proportion across regions, peaking in EC (0.48 × 10⁶ particles/mL) (Table 2).

Regional community structures displayed distinct configurations. Coastal regions were more heterogeneous: YS exhibited a relatively balanced structure with substantial V2 (18.65%) and V3 (13.30%) contributions, while ES showed pronounced V1 dominance (79.83%). Oceanic regions demonstrated lower total abundances but more even subcluster distributions, particularly in KCTWWC and EC with elevated V3 proportions (15.97% and 15.13%, respectively). STCC exhibited the most consistent distribution pattern with moderate V1 dominance (69.84%) (Table 2, Figure A3).

**Figure 4:**
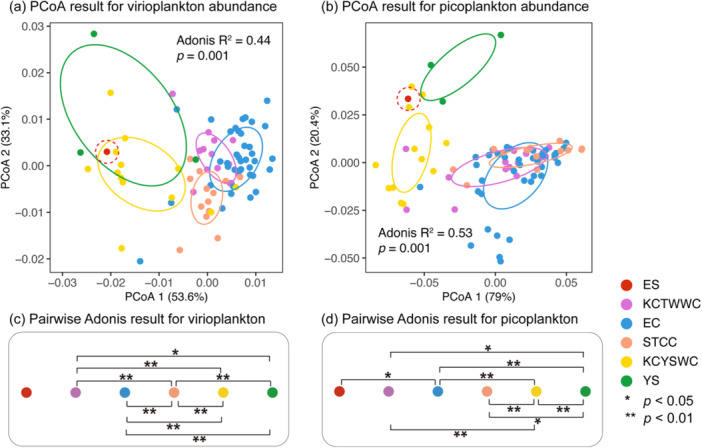
Principal coordinates analysis (PCoA) and pairwise Adonis results showing virioplankton and picoplankton community dissimilarity across regions. (a) PCoA ordination of virioplankton abundance data; (b) PCoA ordination of picoplankton abundance data. The confidence ellipses are based on standard deviation for each group. Note that ES (single data point) is indicated by a manually added dashed circle. (c and d) Pairwise Adonis analysis result for virioplankton and picoplankton abundance. Statistically significant differences between regions were marked as *(*p* < 0.05) or **(*p* < 0.01). Colored dots represent data set from different regions.

PCoA revealed clear spatial segregation of virioplankton communities, with the first two axes explaining 53.6% and 33.1% of the total variance (Figure 4a). Coastal regions (KCYSWC, YS) formed distinct clusters separate from oceanic regions (KCTWWC, EC, STCC). Pairwise Adonis tests confirmed significant interregional differences, most notably between the YS and oceanic clusters (Figure 4c), highlighting substantial spatial heterogeneity in virioplankton abundance.

### 3.3 Abundance and Distributions of Picoplankton

**Figure 5:**
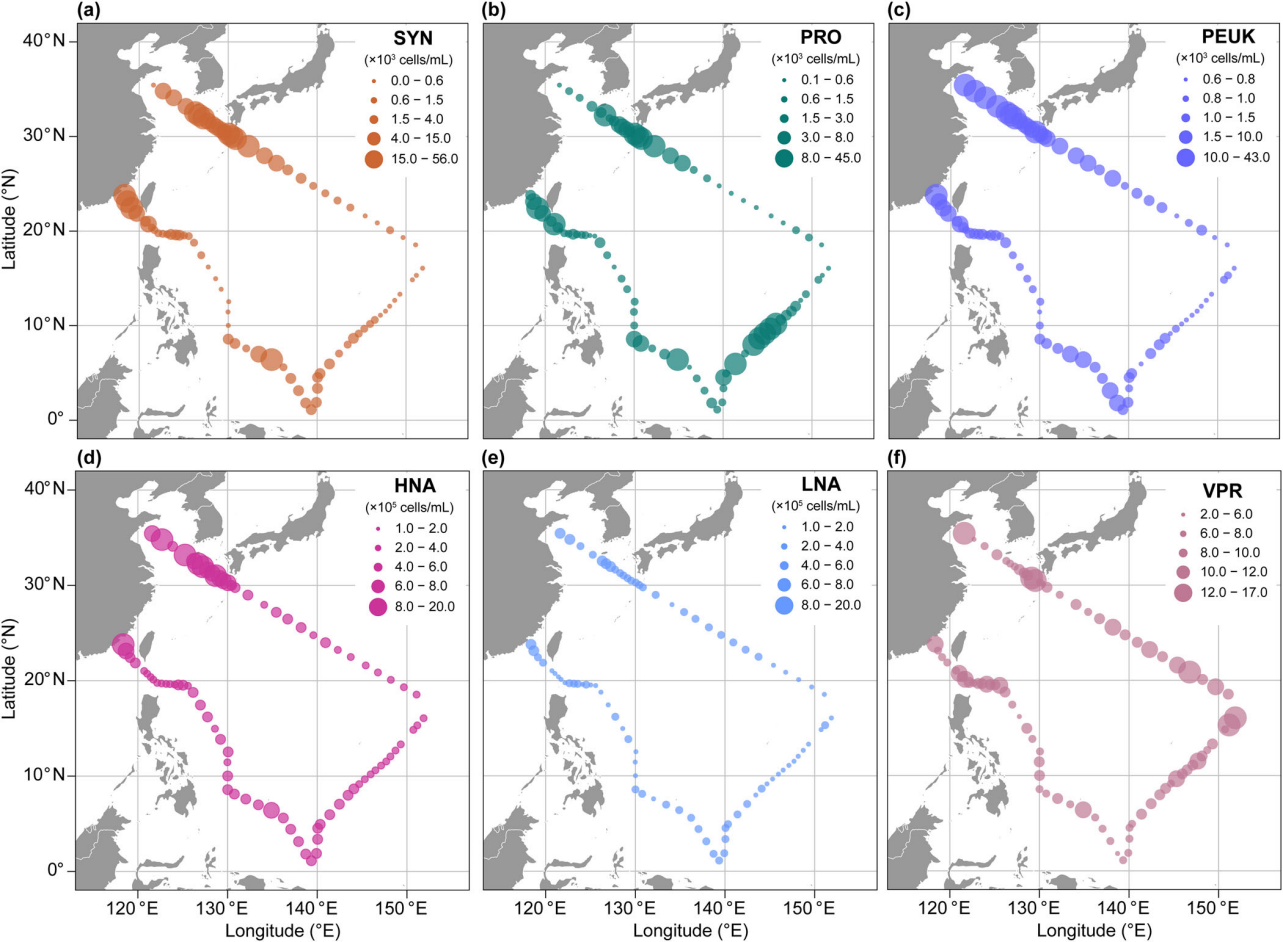
Spatial distribution of picoplankton abundance and virus‐to‐prokaryote ratio (VPR) in the surface waters of the Northwest Pacific. (a) Synechococcus (SYN); (b) Prochlorococcus (PRO); (c) Picoeukaryotes (PEUK); (d) High nucleic acid content heterotrophic prokaryotes (HNA); (e) Low nucleic acid content heterotrophic prokaryotes (LNA); (f) Virus‐to‐prokaryotes ratio (VPR).

The abundance and distribution of picoplankton, the principal hosts of virioplankton, were also investigated. Mean abundances of SYN, PRO, and PEUK were 6.90 ± 11.91 × 10^3^ cells/mL, 5.09 ± 8.87 × 10^3^ cells/mL, and 4.36 ± 7.86 × 10^3^ cells/mL, respectively. SYN and PEUK demonstrated pronounced coastal‐oceanic gradients, with substantially higher abundances in coastal regions (Figure 5a,c), and large standard deviations indicating pronounced regional variability (Table 2). For example, SYN abundance in KCYSWC (22.98 × 10³ cells/mL) was an order of magnitude higher than in EC (2.14 × 10³ cells/mL). PEUK showed even greater variation, with ES abundance (34.08 × 10^3^ cells/mL) exceeding EC levels (1.07 × 10^3^ cells/mL) by more than 30‐fold. In contrast, PRO exhibited a more complex pattern (Figure 5b), with high abundance in both coastal KCYWWC (10.21 × 10^3^ cells/mL) and oceanic EC (5.72 × 10^3^ cells/mL), while remaining sparse in STCC and YS ( ~ 1 × 10^3^ cells/mL).

The distribution of HP, including HNA and LNA, also displayed strong spatial heterogeneity (Figure 5d,e). Mean abundances were 5.07 ± 2.27 × 10⁵ cells/mL for HNA and 2.38 ± 0.80 × 10⁵ cells/mL for LNA, with HNA consistently about twice as abundant as LNA. Both groups followed similar trends, with elevated abundances in nutrient‐rich coastal waters. HNA peaked in YS (10.51 × 10⁵ cells/mL) and reached minimum in STCC (3.88 × 10⁵ cells/mL), while LNA showed maxima in ES (4.10 × 10⁵ cells/mL) and minima in STCC (2.03 × 10⁵ cells/mL), reflecting their affinity for nutrient‐rich environments.

PCoA analysis for picoplankton abundance also revealed clear regional clustering, with the first two axes explaining 79.0% and 20.4% of the total variance, respectively (Figure 4b). Regional differentiation was more pronounced for picoplankton (Adonis *R*² = 0.53, *p* = 0.001) than for virioplankton (*R*² = 0.44, *p* = 0.001). Pairwise Adonis analysis confirmed significant interregional differences, especially between KCYSWC, YS, and EC (Figure 4d).

The virus‐to‐prokaryotes ratio (VPR), calculated as total VLPs abundance (sum of V1–V4) relative to total heterotrophic prokaryote abundance (HNA + LNA), averaged 8.74 ± 2.13 (Table 2). VPR remained relatively stable across regions (Figure 5f), ranging from 8.28 in EC to 10.46 in ES, although individual stations showed greater variation from 5.36 (Stn. 33 in EC) to 16.94 (Stn. 54 in EC).

### 3.4 Phenotypic Diversity of Virioplankton

**Figure 6:**
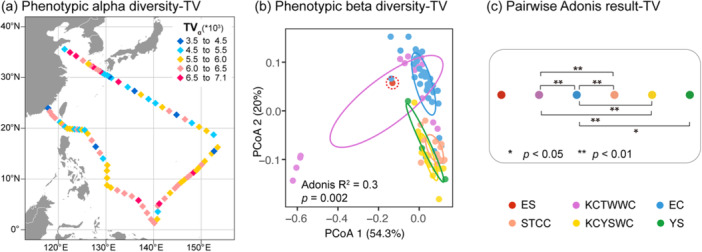
Phenotypic diversity patterns of total VLPs (TV) across the Northwest Pacific. (a) Spatial distribution of phenotypic alpha diversity (inverse Simpson index). (b) Principal coordinates analysis (PCoA) of phenotypic beta diversity based on Bray‐Curtis dissimilarities. The confidence ellipses are based on standard deviation for each group. Note that ES (single data point) is indicated by a manually added dashed circle. (c) Results of pairwise Adonis analysis showing regional differences in beta diversity. Statistically significant differences between regions were marked as *(*p* < 0.05) or **(*p* < 0.01). Colored dots in (b) and (c) represent data set from different regions.

**Figure A4:**
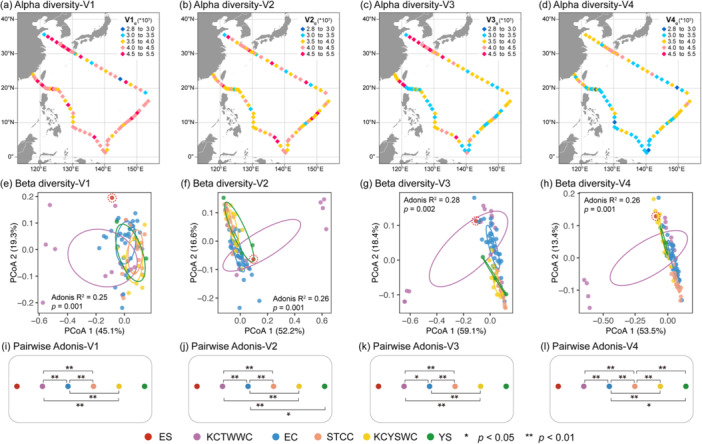
Phenotypic diversity patterns of four viral subclusters (V1–V4) across the Northwest Pacific. (a–d) Spatial distribution of phenotypic alpha diversity (inverse Simpson index) of subclusters V1–V4. (e–h) Principal Coordinates Analysis (PCoA) of phenotypic beta diversity of subclusters V1–V4, based on Bray‐Curtis dissimilarity. The confidence ellipses are based on standard deviation for each group. Note that ES (single data point) is indicated by a manually added dashed circle. (i–l) Results of pairwise Adonis analysis showing regional differences in beta diversity of subclusters V1–V4. Statistically significant differences between regions were marked as *(*p* < 0.05) or **(*p* < 0.01). Colored dots in (e–l) represent data set from different regions.

The phenotypic alpha diversity (inverse Simpson index) showed distinct spatial patterns for TV and its four subclusters. TV alpha diversity (TV_α_) reached its maximum in EC and YS, was moderate in KCTWWC and KCYSWC, and was lowest in ES and STCC (Figure 6a). This pattern largely mirrored that of Subcluster V1 (V1_α_), suggesting its dominant influence on overall viral diversity (Figure A4). While V2_α_ maintained relatively stable diversity across regions, V3_α_ and V4_α_ peaked in KCYSWC and declined toward EC, STCC, and ES. A consistent decline from V1_α_ to V4_α_ indicated progressively simpler community organization among higher subclusters (Figure A4).

Beta diversity analysis revealed significant regional differences in total viral community composition (Adonis *R*² = 0.30, *p* = 0.002). PCoA ordination showed distinct spatial clustering, with the first two axes explaining 54.3% and 20% of total variation (Figure 6b). Pairwise Adonis tests further confirmed most regional contrasts (*p* < 0.01, Figure 6c), emphasizing clear regional differentiation. Notably, Stns. 4–7 in the Luzon Strait (KCTWWC) formed a distinct outlier cluster (Figure 6b), reflecting a distinct community composition relative to neighboring stations. This distinctiveness was also visible in alpha diversity patterns (Figure 6a), suggesting that both community structure and diversity were affected by localized environmental and biological factors.

Individual viral subclusters displayed contrasting regional differentiation in beta diversity (Adonis *R*² = 0.25 ~ 0.28, *p* ≤ 0.001), highlighting their variable environmental sensitivities. Subcluster V1 showed the most pronounced regional distinctions, suggesting greater environmental sensitivity. In contrast, V2, V3, and V4 displayed weaker clustering. Among them, V3 and V4 displayed grouping distinct from V1 and V2. The consistent outlier position of the Luzon Strait stations across all subclusters underscores the unique ecological setting of this region (Figure A4). These results collectively indicate that different viral subclusters respond differently to regional environmental and biological gradients, shaping the complex regional mosaic of virioplankton communities.

### 3.5 Relationships Among Virioplankton and Environmental and Biological Variables

**Figure 7:**
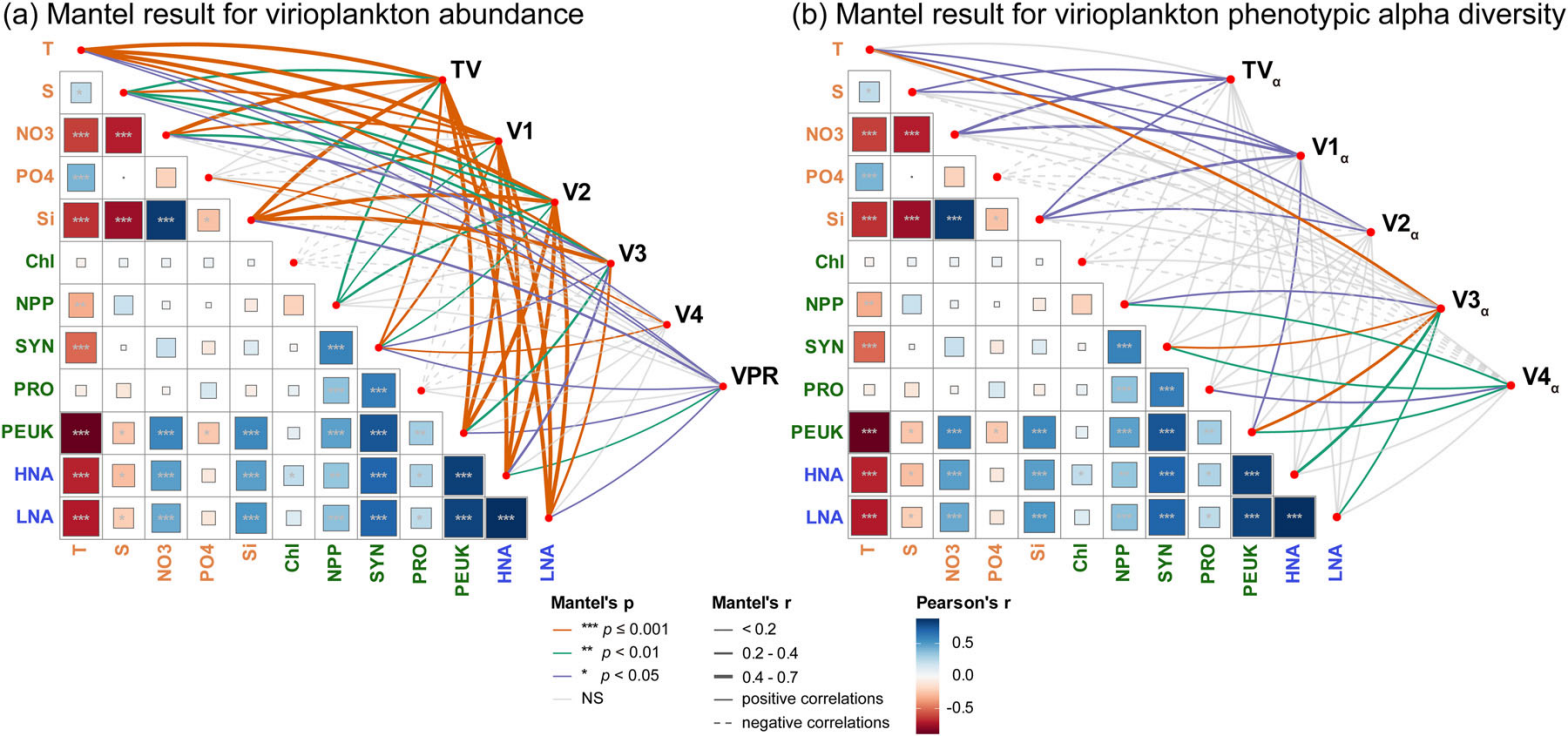
Mantel test results for virioplankton abundance and phenotypic alpha diversity. TV, V1, V2, V3, V4, virioplankton abundance; VPR, virus‐to‐prokaryotes ratio; TV_α_, V1_α_, V2_α_, V3_α_, V4_α_, phenotypic alpha diversity. Environmental parameters: T, temperature; S, salinity; NO3, nitrate; PO4, phosphate; Si, dissolved silicate. Autotrophic biological parameters: Chl, in situ chlorophyll fluorescence; NPP, net primary production; SYN, Synechococcus abundance, PRO, Prochlorococcus abundance, PEUK, picoeukaryotes abundance. Heterotrophic biological parameters: HNA, high nucleic acid content heterotrophic prokaryotes abundance; LNA, low nucleic acid content heterotrophic prokaryotes abundance. Line colors represent significance levels (Mantel's *p*), while line thickness indicates the strength of correlations (Mantel's *r*). Positive and negative correlations are represented by solid and dashed lines, respectively. The heatmap squares show Pearson's correlation coefficients (Pearson's *r*) between environmental variables and microbial groups, with colors indicating the direction and magnitude of the correlations, as shown in the color legend.

Mantel test revealed complex relationships between viral populations and environmental‐biological variables. TV and subclusters V1–V3 abundance showed strong associations with temperature, nitrate, and silicate (Figure 7a, orange lines, *p* ≤ 0.001), as well as with both autotrophic (SYN and PEUK) and heterotrophic (HNA and LNA) hosts. By contrast, V4 showed considerably weaker correlations, suggesting distinct ecological controls. For viral phenotypic alpha diversity, although significant correlations were observed with temperature, nutrients, and host abundance, these associations were generally less pronounced than those for viral abundance (Figure 7b), implying that viral diversity is modulated by more complex regulatory mechanisms beyond environmental variables and host abundance.

**Figure 8:**
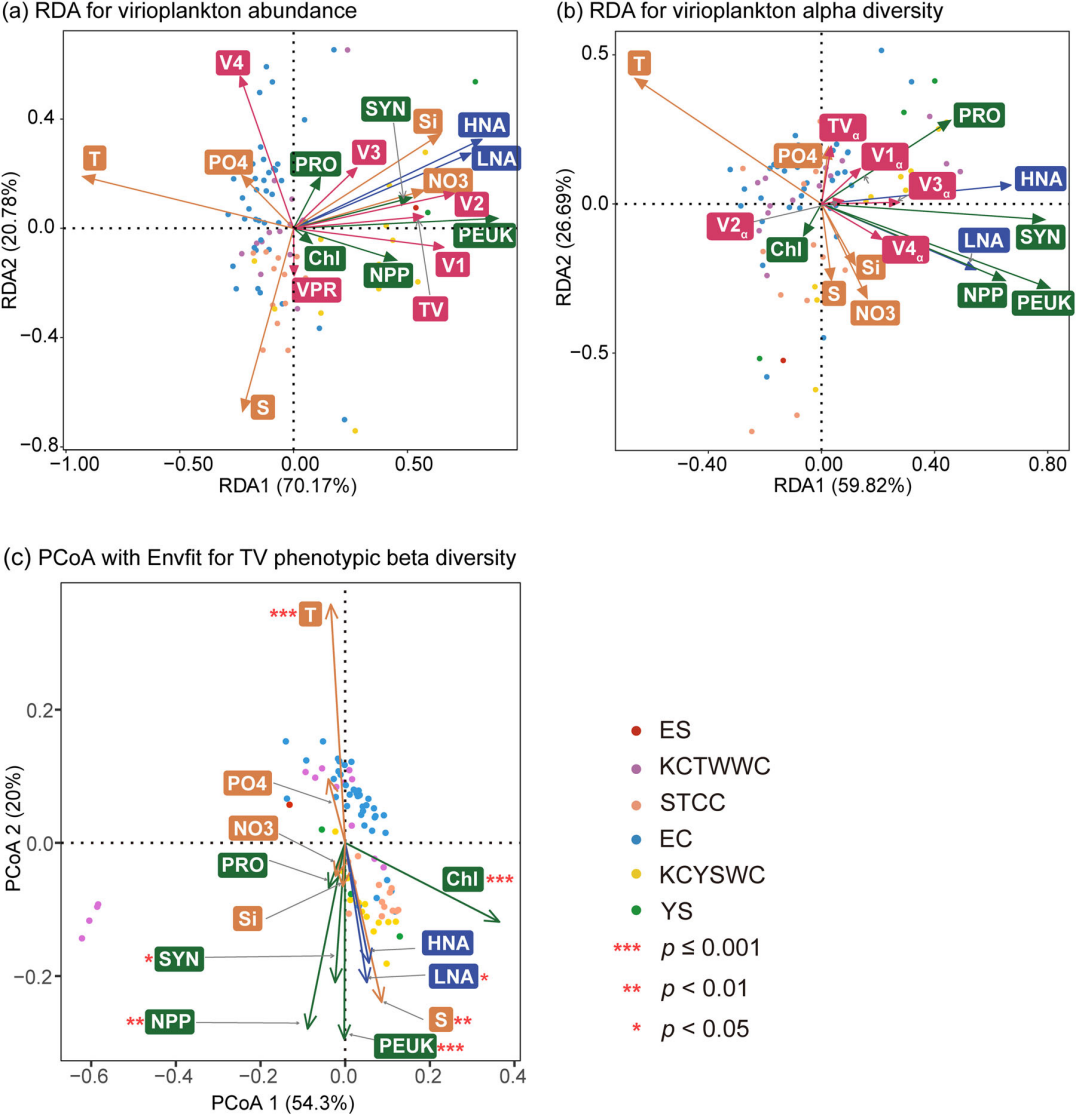
Analysis of the regulation of virioplankton community structure by environmental and biological factors across sampling stations. (a) Redundancy analysis (RDA) showing the relationship between environmental and biological factors and virioplankton abundance. (b) RDA showing the relationship between environmental and biological factors and virioplankton phenotypic alpha diversity. (c) Principal coordinates analysis (PCoA) plot of phenotypic beta diversity of total VLPs (TV), with Environmental Fit (Envfit) overlaid, examining the regulation of beta diversity by various environmental and biological factors. TV, V1, V2, V3, V4, virioplankton abundance; VPR, virus‐to‐prokaryotes ratio; TV_α_, V1_α_, V2_α_, V3_α_, V4_α_, phenotypic alpha diversity. Environmental parameters: T, temperature; S, salinity; NO3, nitrate; PO4, phosphate; Si, dissolved silicate. Autotrophic biological parameters: Chl, in situ chlorophyll fluorescence; NPP, net primary production; SYN, Synechococcus abundance, PRO, Prochlorococcus abundance, PEUK, picoeukaryotes abundance. Heterotrophic biological parameters: HNA, high nucleic acid content heterotrophic prokaryotes abundance; LNA, low nucleic acid content heterotrophic prokaryotes abundance. Statistically significant factors on panel (c) were marked as *(*p* < 0.05), **(*p* < 0.01), or ***(*p* ≤ 0.01). Colored dots represent data set from different regions.

Redundancy analyses (RDA) demonstrated that environmental and biological factors jointly explained substantial proportions of variance in viral abundance (90.95%) and phenotypic alpha diversity (86.51%). In the abundance RDA, temperature and salinity loaded negatively along RDA1 and RDA2, in contrast with nutrients and biological variables (Figure 8a). TV abundance correlated moderately with microbial hosts, particularly PEUK and SYN in cooler waters. Different subclusters exhibited distinct ecological affinities: V1 was most strongly related to primary production and PEUK, V2 and V3 were associated with heterotrophic prokaryotes in nutrient‐rich waters, while V4 showed a unique distribution pattern characterized by strong negative association with salinity. Notably, VPR displayed weak associations overall, implying that virus–host interactions extend beyond simple linear relationships.

RDA of phenotypic diversity revealed different structuring patterns (Figure 8b). Temperature showed a strong negative loading on RDA1, while salinity and nutrients (NO3, Si) loaded negatively on RDA2. Three primary clusters were evident: (1) TV_α_, V1_α_, and V2_α_ clustered together, correlating positively with PO4 and PRO; (2) V3_α_ aligned with HNA; and (3) V4_α_ oriented toward PEUK, NPP, and LNA. These differences indicate that drivers of phenotypic diversity may differ from those controlling viral abundances.

Environmental Fitting (Envfit) analysis identified chlorophyll as the strongest correlate of viral phenotypic beta diversity (*R*² = 0.300, *p* ≤ 0.001), followed by temperature (*R*² = 0.264, *p* ≤ 0.001) and NPP (*R*² = 0.175, *p* < 0.01) (Figure 8c). PEUK, salinity, LNA, and SYN also showed significant but weaker correlations (Table A1). Subclusters varied in their environmental sensitivities: V1 correlated mainly with chlorophyll and heterotroph abundance. V2 and V3 displayed similar patterns, primarily correlated with chlorophyll, NPP, and physical factors. V4 showed the most complex correlation pattern, significantly associated with multiple factors, including heterotroph abundance, PEUK, chlorophyll, temperature, and NPP. Notably, chlorophyll consistently emerged as the strongest and most universal correlate of viral phenotypic beta diversity across all subclusters, while nutrients showed no significant influence, suggesting that host availability and productivity, rather than nutrient supply, primarily shape viral community organization.

## 4 Discussion

In the Northwest Pacific, total viral abundances ranged from 3.69 × 10⁶ to 17.09 × 10⁶ particles/mL, consistent with previous reports from the Pacific Ocean (Liang et al. 2017; Wei et al. 2018; Yang et al. 2010) and other oceanic regions (De Corte et al. 2016; Liang et al. 2014; Magiopoulos and Pitta 2012). While earlier studies typically identified two to three viral subclusters in the region (Liang et al. 2017; Wei et al. 2018; Yang et al. 2010), four major subclusters were identified throughout our survey, with an additional one detected in the Yellow Sea coastal waters (Stn. 84). This enhanced resolution provides evidence for previously unrecognized viral diversity patterns.

Regional variations in viral subcluster distributions highlighted the influence of both environmental and biological factors on viral community structure. Correlation analyses revealed strong associations between specific viral subclusters and environmental or biological parameters. Of particular interest, phenotypic diversity analysis revealed unique viral community structures in the Luzon Strait (Stns. 4–7). These findings highlight the complex interactions between environmental gradients and biological processes that shape virioplankton communities, advancing our understanding of their ecological roles across diverse marine ecosystems.

### 4.1 Methodological Advances and Implications of Phenotypic Diversity Analysis

Traditional sequencing‐based approaches, although powerful, face notable limitations in capturing the full spectrum of viral diversity. Many viral sequences remain taxonomically unassigned due to the vast unknowns of the global virosphere (Gregory et al. 2019; Hurwitz and Sullivan 2013). Moreover, multiple processing steps, such as viral concentration, nucleic acid extraction, and amplification, can introduce significant biases (Hurwitz et al. 2013; Kim and Bae 2011), potentially leading to skewed community profiles. These challenges often result in underestimation or misinterpretation of viral diversity.

Flow cytometric diversity analysis, first introduced by Li (1997), quantifies biodiversity based on the bio‐optical characteristics of individual particles. These include size, structure, morphology, and nucleic acid content, together forming a phenotypic fingerprint of the community. The phenotypic diversity framework Phenoflow (Props et al. 2016) integrates these characteristics into diversity indices that reflect community complexity while minimizing sample manipulation. This method enables near‐direct measurement without cell lysis or nucleic acid extraction (Props et al. 2018) and shows moderate to strong correlations with genotypic diversity derived from traditional amplicon sequencing methods (Heyse et al. 2021; Props et al. 2016; Saini et al. 2022; Zhong et al. 2020), supporting its reliability as a complementary approach.

Conventional flow cytometers often struggle to resolve viral populations due to their low scatter and fluorescence intensities (below 10¹ arbitrary units). However, the integration of violet lasers and APDs has significantly improved signal resolution, enabling better discrimination from background noise and clearer separation of viral subclusters (Zhao et al. 2023). This technological advance facilitates the effective application of Phenoflow to virioplankton studies, providing new insights to explore viral structural and functional complexity.

Our methodological framework, which integrates advanced FCM with Phenoflow analysis, revealed previously undetected patterns in viral community structure. This represents an extension of Phenoflow beyond bacterial and phytoplankton applications (Wijaya et al. 2023). While this approach does not provide detailed taxonomic resolution, the extensive spatio‐temporal coverage achieved through FCM provides compensatory advantages. In the Luzon Strait, for example, traditional environmental and biological parameters (e.g., temperature, salinity, nutrients, chlorophyll, NPP, and pico‐ and virioplankton abundance) showed no significant deviations relative to adjacent stations, yet phenotypic alpha and beta diversity revealed distinct anomalies in viral communities. Such cryptic patterns, undetectable through conventional approaches, demonstrate the enhanced sensitivity of phenotypic diversity analysis in capturing subtle community shifts.

The integration of phenotypic diversity analysis into marine viral ecology provides a scalable hierarchical framework for investigating viral communities. As a high‐throughput screening tool, it can efficiently identify diversity hotspots and community anomalies across large spatial scales. These identified regions of interest, such as the cryptic diversity hotspot in the Luzon Strait, can subsequently be targeted for flow virometry‐based sorting and multi‐omic analyses to elucidate underlying ecological mechanisms.

Beyond screening, this framework also offers potential for ecological monitoring. Machine learning‐based automation of FCM data (Özel Duygan and van der Meer 2022; Rubbens et al. 2023) could allow real‐time detection of community shifts in response to environmental and biological stressors, such as ocean acidification, eutrophication (Malits et al. 2021), hypoxia (Jurgensen et al. 2022), or algal blooms (Han et al. 2023). When anomalous fluctuations in viral abundance or diversity are detected, targeted samples can be subjected to flow sorting and multi‐omic analyses to uncover causal mechanisms. This hierarchical framework, integrating high‐throughput screening with detailed molecular characterization, enables both large‐scale monitoring and fine‐scale ecological interpretation of marine viral communities.

### 4.2 Viral Subcluster Dynamics Along the Coastal‐Oceanic Continuum

Previous studies in the Western Pacific revealed varying numbers of viral subclusters, ranging from three to four in a Western Pacific seamount region (Zhao et al. 2020) and up to six in coastal waters such as Jiaozhou Bay (Zhao et al. 2023). Our investigation along the coastal‐oceanic gradient revealed four consistent subclusters across the Northwest Pacific surface waters, with an additional subcluster appearing only at Stn. 84 in the Yellow Sea. This finding suggests that major community transition shifts occur within narrow transitional zones rather than gradually along environmental gradients.

Flow cytometric viral subclusters correspond to distinct ecological functional groups, with fluorescence intensity to some extent reflecting nucleic acid content (Brussaard et al. 2010). Generally, low fluorescence viral (LFV) subcluster primarily consists of bacteriophages, medium fluorescence viral (MFV) subcluster comprises cyanophages and viruses infecting small algae, while high fluorescence viral (HFV) subcluster is typically viruses infecting large algae (Brussaard and Martinez 2008; Larsen et al. 2004; Martínez et al. 2014; Mojica et al. 2016; Payet et al. 2014). Among the subclusters identified in this study, V1 corresponds to LFV, V3 corresponds to MFV, and V4 corresponds to HFV, whereas V2 likely represents a mixture of larger bacteriophages and smaller cyanophages. These associations are supported by the Mantel test and RDA results.

The subcluster distribution patterns exhibited clear coastal‐oceanic differentiation across the Northwest Pacific. Bacteriophage‐dominated V1 (LFV) showed notably higher proportions in coastal regions, consistent with previous studies (Baudoux et al. 2008; Evans et al. 2017). In contrast, V2 and V3 displayed contrasting patterns. V2 (mixed bacteriophages and cyanophages) showed higher proportions in coastal regions, whereas V3 (mainly cyanophages and viruses of small algae) prevailed in oceanic regions. This pattern resembles that reported by Mojica et al. (2016), likely reflecting a transition in host community composition from bacteria‐dominated coastal waters to cyanobacteria‐dominated oceanic zones. V4 (HFV) maintained consistently low abundance across all regions, showing a slight preference for oceanic waters, similar to patterns observed in other oligotrophic systems (Yang et al. 2010).

Additional viral subclusters are often observed during phytoplankton blooms (Brussaard and Martinez 2008; Jacquet et al. 2002; Larsen et al. 2008). However, the detection of V5 at Stn. 84 and the previously reported V5 and V6 in Jiaozhou Bay (Zhao et al. 2023) occurred under non‐bloom conditions. These additional subclusters, characterized by high fluorescence content, likely represent intrinsic components of coastal viral communities rather than bloom‐associated populations.

An intriguing paradox emerged at Stn. 84, where the occurrence of an extra viral subcluster coincided with a decline in total viral alpha diversity compared to the adjacent stations (Figure 6a). The phenotypic alpha diversity (measured by the inverse Simpson index) integrates both subcluster richness and their relative evenness (Props et al. 2016). Despite V5 representing a novel subcluster, its extremely low abundance (0.18 × 10⁶ particles/mL, 1.05% of total abundance) compared with V1 (10.14 × 10⁶ particles/mL, 59.34% of total) created a highly uneven distribution. This skewness implies that the addition of V5, instead of enhancing evenness, introduced an imbalance that reduced the overall diversity index.

### 4.3 Environmental and Biological Drivers of Viral Community Structure

Large‐scale viral biogeography is heavily influenced by ocean currents, as demonstrated in previous studies (Brum et al. 2015; Yang et al. 2014). Similarly, in the Northwest Pacific, water masses serve as key organizing forces for virioplankton community structure. Variations in viral abundance and diversity reflect both environmental gradients and host availability across regions defined by distinct water masses. Ordination analyses revealed pronounced regional patterns, with coastal waters forming tighter clusters due to compositional similarity, while oceanic waters displayed greater heterogeneity. Comparable regional differentiation has also been reported in the Southern Ocean, where distinct viral communities were shaped by contrasting environmental and biological conditions (Sotomayor‐Garcia et al. 2020).

In the Northwest Pacific, viral abundance peaked in nutrient‐rich coastal regions, coinciding with elevated heterotrophic prokaryote densities. Oceanic waters exhibited lower abundances but greater compositional variability, consistent with host transitions toward cyanobacteria and picoeukaryotes. Similar coastal‐oceanic contrasts have been documented in the Mediterranean Sea (Magiopoulos and Pitta 2012), Prydz Bay and the adjacent Southern Ocean (Liang et al. 2016), and the Northestern Pacific (Culley and Welschmeyer 2002), suggesting a recurring biogeographic pattern in marine viral ecology.

Interestingly, despite marked variations in absolute abundance and community composition, VPR remained relatively stable across regions in our survey. This stability, together with consistent community clustering, suggests tightly coupling between viruses and their microbial hosts. Such coupling likely reflects the co‐evolutionary dynamics that maintain equilibrium between viral replication and host availability (Parikka et al. 2017), even under varying environmental conditions.

Spatial distribution of virioplankton is shaped by multiple factors, with host abundance, chlorophyll levels, and temperature as primary drivers (Mojica and Brussaard 2014; Wommack and Colwell 2000). The strong correlation between temperature and viral community structure observed in our study confirms temperature as a key environmental driver, consistent with findings from field studies (Bettarel et al. 2009; Finke et al. 2017; Li et al. 2014; Maurice et al. 2013) and modeling (Winter et al. 2012). Among biological drivers, autotrophic factors such as chlorophyll, NPP, and PEUK exhibited stronger correlation strength with viral assemblages than heterotrophic factors (HNA and LNA). This pattern, consistent with observations from Prydz Bay (Liang et al. 2016), highlights the role of cyanophages and algal viruses in structuring viral communities in these regions. These findings, combined with regional clustering in viral diversity, align with observations from the Southern Ocean (Sotomayor‐Garcia et al. 2020), where environmental gradients and host availability jointly shape the viral community.

A particularly notable feature observed in this study was the distinct viral community structure in the Luzon Strait (Stns. 4–7), characterized by unique clustering in phenotypic diversity compared to the adjacent stations. This region, shaped by Kuroshio intrusions, monsoonal influences, and steep topography, experiences strong localized upwelling (Guo et al. 2017) and intense mesoscale eddy activities (Li et al. 1998). Such hydrodynamic conditions can stimulate local biological processes, enhance primary productivity, and trigger seasonal phytoplankton blooms (Guo et al. 2017; Tang et al. 1999). Mixing processes, such as upwelling and turbulence, are known to enhance viral production and abundance by increasing nutrient fluxes and stimulating microbial host activity (He et al. 2009; Muck et al. 2014; Paterson et al. 2012; Winter et al. 2018).

In the upwelling region of the Luzon Strait, Liang et al. (2017) reported slightly higher LFV abundances in the upper 75 m, whereas HFV showed little vertical variation. Enhanced subsurface (25–50 m) SYN and PEUK abundances and elevated surface HP (0–25 m) were also noted. In a “cryptic” upwelling system in South Australian waters, where upwelled waters reached only the Deep Chlorophyll Maximum layer, viral subcluster V3 was more abundant in upwelling‐influenced sites, while V1 and V2 remained stable. Host groups such as HNA1, HNA3, PRO, and PEUK also increased in the upwelling‐affected regions, while LNA, HNA2, and SYN showed minimal change (Paterson et al. 2013).

Our surface‐based observations revealed a distinct but related pattern: viral diversity indices differed significantly between upwelling‐affected and adjacent non‐upwelling stations. However, unlike earlier studies, no significant differences were detected in viral or picoplankton abundances. This may indicate weak or intermittent upwelling that was sufficient to alter community structure but not strong enough to significantly impact bulk abundances. Alternatively, rapid surface mixing or advection at the surface might have masked abundance gradients while preserving compositional signatures. Future depth‐resolved sampling and time‐series observations will be crucial for elucidating the link between upwelling intensity and viral community dynamics.

## 5 Conclusion

This study provides new insights into the biogeography of virioplankton in the Northwest Pacific, highlighting the complexity of viral communities through the integration of advanced FCM and phenotypic diversity analyses. By resolving four consistent viral subclusters across oceanic and coastal waters and detecting an additional subcluster in the Yellow Sea, we revealed previously underappreciated patterns of viral diversity in these regions. Phenotypic diversity analysis further revealed cryptic patterns, including a distinct community structure in the Luzon Strait, demonstrating its capability to detect ecological variations that traditional sequencing or environmental variables may easily overlook.

Environmental gradients, including temperature, chlorophyll, and picoplankton abundance, emerged as the primary drivers of viral community structure, underscoring the tight coupling between virioplankton and their microbial hosts across contrasting marine environments. These findings contribute to a broader understanding of how mesoscale oceanographic features and environmental factors jointly shape microbial and viral community dynamics in the marine ecosystem.

## Author Contributions

**Yuan Zhao:** conceptualization (lead), methodology (lead), formal analysis (lead), visualization (lead), writing – original draft (lead), writing – review and editing (lead), funding acquisition (equal). **Yanchu Zhao:** investigation (lead), formal analysis (lead), writing – original draft (equal), writing – review and editing (equal). **Yi Dong:** formal analysis (supporting), visualization (supporting), writing – original draft (supporting), writing – review and editing (supporting). **Xiaoxia Sun:** formal analysis (supporting), writing – original draft (supporting), writing – review and editing (supporting). **Wuchang Zhang:** writing – original draft (supporting), writing – review and editing (supporting), funding acquisition (equal). **Li Zhao:** conceptualization (lead), formal analysis (supporting), visualization (supporting), writing – original draft (lead), writing–review and editing (lead). **Gérald Grégori:** methodology (lead), formal analysis (supporting), writing – original draft (supporting), writing – review and editing (supporting), funding acquisition (equal).

## Ethics Statement

The authors have nothing to report.

## Conflicts of Interest

The authors declare no conflicts of interest.

## Data Availability

The data sets used and analyzed during the current study are publicly available in the figshare repository at: https://doi.org/10.6084/m9.figshare.30617255.

## 1

**Table A1:** Relationships between environmental/biological variables and viral community beta diversity based on PCoA ordination with environmental fitting.

Variable	TV		V1		V2		V3		V4	
	*R* ^2^	Sig	*R* ^2^	Sig	*R* ^2^	Sig	*R* ^2^	Sig	*R* ^2^	Sig
T	0.264	***	0.053	NS	0.082	NS	0.112	*	0.178	**
S	0.132	**	0.056	NS	0.110	*	0.118	*	0.051	NS
Chl	0.300	***	0.311	***	0.285	***	0.318	***	0.217	***
NO3	0.006	NS	0.025	NS	0.002	NS	0.002	NS	0.075	NS
PO4	0.022	NS	0.009	NS	0.013	NS	0.019	NS	0.025	NS
Si	0.009	NS	0.001	NS	0.002	NS	0.001	NS	0.079	NS
NPP	0.175	**	0.022	NS	0.117	**	0.108	*	0.127	**
SYN	0.091	*	0.055	NS	0.014	NS	0.064	NS	0.080	*
PRO	0.012	NS	0.006	NS	0.016	NS	0.023	NS	0.023	NS
PEUK	0.177	***	0.041	NS	0.039	NS	0.071	NS	0.214	***
HNA	0.073	NS	0.085	*	0.015	NS	0.035	NS	0.222	**
LNA	0.095	*	0.072	*	0.027	NS	0.047	NS	0.180	**

*Note:* Values show goodness‐of‐fit (*R*²) and significance levels (Sig, ***: *p* ≤ 0.001; **: *p* < 0.01; ***: *p* < 0.05; NS, not significant). TV: total VLPs; V1–V4: viral subclusters. Environmental variables: T, temperature; S, salinity; NO3, nitrate; PO4, phosphate; Si, dissolved silicate. Biological variables: Chl, in situ chlorophyll fluorescence; NPP, net primary production; SYN, *Synechococcus* abundance, PRO, *Prochlorococcus* abundance, PEUK, picoeukaryotes abundance; HNA, high nucleic acid content heterotrophic prokaryotes abundance; LNA, low nucleic acid content heterotrophic prokaryotes abundance.
